# Application of Physiologically Based Pharmacokinetic Modeling in Preclinical Studies: A Feasible Strategy to Practice the Principles of 3Rs

**DOI:** 10.3389/fphar.2022.895556

**Published:** 2022-05-12

**Authors:** Yawen Yuan, Qingfeng He, Shunguo Zhang, Min Li, Zhijia Tang, Xiao Zhu, Zheng Jiao, Weimin Cai, Xiaoqiang Xiang

**Affiliations:** ^1^ Department of Clinical Pharmacy and Pharmacy Administration, School of Pharmacy, Fudan University, Shanghai, China; ^2^ Department of Pharmacy, Shanghai Children’s Medical Center, School of Medicine, Shanghai Jiao Tong University, Shanghai, China; ^3^ Department of Pharmacy, Shanghai Chest Hospital, Shanghai Jiao Tong University, Shanghai, China

**Keywords:** physiologically based pharmacokinetic modeling, 3Rs, preclinical studies, alternative for animal experiments, bottom-up model

## Abstract

Pharmacokinetic characterization plays a vital role in drug discovery and development. Although involving numerous laboratory animals with error-prone, labor-intensive, and time-consuming procedures, pharmacokinetic profiling is still irreplaceable in preclinical studies. With physiologically based pharmacokinetic (PBPK) modeling, the *in vivo* profiles of drug absorption, distribution, metabolism, and excretion can be predicted. To evaluate the application of such an approach in preclinical investigations, the plasma pharmacokinetic profiles of seven commonly used probe substrates of microsomal enzymes, including phenacetin, tolbutamide, omeprazole, metoprolol, chlorzoxazone, nifedipine, and baicalein, were predicted in rats using bottom-up PBPK models built with *in vitro* data alone. The prediction’s reliability was assessed by comparison with *in vivo* pharmacokinetic data reported in the literature. The overall predicted accuracy of PBPK models was good with most fold errors within 2, and the coefficient of determination (R^2^) between the predicted concentration data and the observed ones was more than 0.8. Moreover, most of the observation dots were within the prediction span of the sensitivity analysis. We conclude that PBPK modeling with acceptable accuracy may be incorporated into preclinical studies to refine *in vivo* investigations, and PBPK modeling is a feasible strategy to practice the principles of 3Rs.

## 1 Introduction

The pharmacokinetics study, including examining absorption, distribution, metabolism, and excretion (ADME) profiles of therapeutic agents, plays a vital role in drug discovery and development (Prentis et al., 1988). Because of poor extrapolation from *in vitro* to *in vivo* efficacy, pharmacokinetics profiling processes are routinely implemented in the pharmaceutical industry for early preclinical optimization (Lin and Lu, 1997). However, such processes commonly involve error-prone, labor-intensive, and time-consuming procedures. Not to mention ethics and the welfare of laboratory animals. It was estimated that more than 100 million laboratory animals were sacrificed for biomedical research annually (Taylor and Alvarez, 2019). Therefore, William Russel and Rex Burch proposed the 3Rs principle (replacement, reduction, and refinement) in 1959 (Vitale et al., 2009; Wachsmuth et al., 2021), attempting to reduce animal use. The 2010 EU Directive states that animals have intrinsic values that need to be respected and that animal experiments should be carefully evaluated in biomedical research, with animal welfare considerations a top priority. Currently, the 3Rs have evolved into basic requirements for researchers to comply with based on animal welfare legislation. Several non-animal testings, including *in vitro* and *in silico* approaches describing the ADME properties, have also been developed to achieve high-throughput screening in drug development. However, unlike understanding the all-inclusive fate of compounds in the body through animal experiments, these conventional *in vitro* methods generally only cover a single-ADME process (Pelkonen and Turpeinen, 2007; Cascone et al., 2016). For example, the commonly used *in vitro* methods for studying drug absorption (Irvine et al., 1999; Verhoeckx et al., 2015; Dargó et al., 2019) or metabolism (Raunio et al., 2004; Hariparsad et al., 2006; Pelkonen and Turpeinen, 2007; van de Kerkhof et al., 2007), such as artificial biofilm models, cell models, and microsomal experiments. Similarly, *in silico* approaches, including the quantitative structure–activity relationship construction model (QSAR), were generally applied to predict the individual biological activity of candidate compounds such as apparent permeability (*P*_app_), plasma protein binding rate, and apparent volume of distribution (Vd_ss_) in the early drug discovery process (Ekins et al., 2000; Yamashita and Hashida, 2004; Lombardo et al., 2017). Despite the abundant data sources, a key challenge remains in correlating the *in vitro* results of ADME features to establish *in vivo* models to reflect the overall disposal.

PBPK modeling was raised back in 1937 and initially applied in predicting the distribution of environmental compounds in mammalian tissues (Lindstrom et al., 1974), and further gradually used for drug exposure prediction, dose extrapolation, and safety assessment (Andersen et al., 1987; Haddad et al., 2001; Meek et al., 2013; Li et al., 2021). PBPK modeling is a mathematical method following the material balance principle to predict the time course of xenobiotic levels in plasma and tissues based on the physiochemical and pharmacokinetic parameters of compounds (Nestorov, 2003). Tremendous progress has been made in PBPK modeling during the past decade. In addition to the rapidly gained industrial recognition (Rowland et al., 2011; Miller et al., 2019), PBPK analysis has also become a routine for the regulatory authorities, especially the United States Food and Drug Administration (FDA), upon new drug applications since 2016 (Zhang et al., 2020).

Currently, the PBPK model has been widely used in various stages of drug development (Chen et al., 2012; Huang et al., 2013), such as evaluating interspecies differences, drug–drug interactions (Jin et al., 2022), targeted tissue exposure, and disease effect (Rostami-Hodjegan and Tucker, 2007; Clewell and Clewell, 2008; Hans and Ursula, 2009; Rietjens et al., 2010; Mielke et al., 2011; Ball et al., 2014). In addition to the top–down modeling, many PBPK models have adopted *in vitro* PK parameters for bottom–up modeling or experimental *in vivo* parameters for “middle-out” approaches as preliminary verification and model optimization (Rostami-Hodjegan and Tucker, 2007; Rietjens et al., 2010; Kostewicz et al., 2014; Templeton et al., 2018; Chang et al., 2019; Umehara et al., 2019). Moreover, given some mechanistic reasons (such as paracellular absorption, active absorption, and targeted transport), many bottom–up PBPK models still require animal data (Wagner, 1981; Clewell and Clewell, 2008). For example, the distribution parameters used in many PBPK models are tissue partition coefficients or steady state distribution parameters from *in vivo* experiments (Harrison and Gibaldi, 1977; Igari et al., 1983; S et al., 2007; T’jollyn et al., 2018). A few PBPK modelings are wholly constructed from *in vitro* parameters without *in vivo* parameters to be fitted and optimized, lacking universal application (Cheng and Ng, 2017). Therefore, constructing a universal PBPK model entirely only with *in vitro* data and the issue of estimating the predicted accuracy in the absence of *in vivo* PK data have caused widespread concern recently (Ellison, 2018; Paini et al., 2019).

To evaluate the feasibility of using PBPK modeling as an alternative for animal experiments, we developed bottom–up PBPK models solely with *in vitro* data using Simcyp^®^ (Sheffield, United Kingdom) to predict the systemic disposition of seven commonly used liver microsomal enzyme probe substrates in rats. The reliability of each prediction was examined with sensitivity analysis. As a practice of 3Rs, this proof-of-concept study will shed light on the refinement of current drug development procedures and may reduce animal usage in drug development.

## 2 Materials and Methods

### 2.1 Model Inputs Collection

Typical probe substrates of cytochrome P450 (CYP) 1A2, CYP2C9, CYP2C19, CYP2D6, CYP2E1, CYP3A4/5, and UDP-glucuronosyltransferase (UGT), namely, phenacetin, tolbutamide, omeprazole, metoprolol, chlorzoxazone, nifedipine, and baicalein were used as model drugs. The *in vitro* parameters were divided into three categories. Physical chemistry and blood binding-related parameters include molecular weight (MW), neutral species octanol: water partition coefficient (log*P*_o:w_), compound type (base/acid/mono/diprotic/ampholyte), negative decadic logarithm of the ionization constant of an acid (p*K*_a_), blood to plasma partition ratio (B/P), and fraction unbound in plasma (*f*_u_). Distribution is affected by the free fraction and the lipid solubility of the drug, which is related to the physiological characteristics mentioned, such as log*P*_o:w_, p*K*_a_, and *f*_u._ Parameters about absorption include apparent permeability coefficients (*P*_app_), polar surface area (PSA), and hydrogen bond donors (HBD). *In vivo* elimination can be extrapolated *via* a well-stirred liver model together with the parallel tube model (Pang and Rowland, 1977) and the dispersion model (Iwatsubo et al., 1997) using *in vitro* metabolic data, including Michaelis–Menten constant for metabolism (*K*_m_), maximum velocity for metabolism (*V*_max_), and intrinsic clearance (CL_int_). Various *in vitro* data of model drugs collected from different literature and databases (DrugBank, PubChem, HSDB, TOXNET, *etc*.) were compiled in Supplementary Table S1, and the units of each parameter were uniformly converted.

### 2.2 Physiologically Based Pharmacokinetic Modeling

PBPK models were constructed with *in vitro* data alone using Simcyp^®^ (Simcyp Rat Version 16, Certara, Sheffield, United Kingdom). The *in vitro* parameters used in the PBPK model are described in Table 1. The first-order one-compartment model was selected, and the gut was considered as a single compartment in this model. The permeability-limited basolateral membrane is assumed to mediate the absorption of drugs from enterocyte to the intestinal interstitial fluid, and effective permeability in rat (*P*_eff_) was calculated based on its relationship with *P*_eff_ in human (Cao et al., 2006), which can be extrapolated from *P*_app,_ obtained from *in vitro* experiments (Sun et al., 2002; Tchaparian et al., 2008) or predicted using the PSA and HBD models (Winiwarter et al., 1998). Initially, our absorption model adopted the PSA and HBD models. A minimal PBPK model was selected to predict the volume of distribution at steady state (*V*_ss_) with *in vitro* parameters of log*P*_o:w_, compound type, and p*K*_a_ using mechanistic model 2 (Poulin and Theil, 2002; Berezhkovskiy, 2004; Rodgers et al., 2005; Rodgers and Rowland, 2007), which is a preset model in Simcyp. The tissue distribution was predicted using *K*_p_ scalar (tissue: plasma partition coefficient) based on a perfusion-limited model. The *K*_p_ scalar was selected as 1 by default in our model. The whole organ metabolic clearance pane was selected in the elimination screen. The liver or intestinal clearance was extrapolated *via in vitro* metabolic data.

**TABLE 1:** *In vitro* parameters used in each module of the PBPK model.

Application module	*In vitro* parameter	Unit	Acquisition method
Physical chemistry and blood binding	MW	g/mol	Calculation
	log*P*_o:w_	—	*In vitro* measurement
	Compound type	Base/acid/mono/diprotic/ampholyte	Physicocheistry property
	p*K*_a_	—	*In vitro* measurement^a^
	B/P	—	*In vitro measurement*
	*f*_u_	—	*In vitro* measurement
Absorption	*P*_app_	10^−6^ cm/s	*In vitro* experimentation^b^
	PSA	Å^2^	Calculation or prediction
	HBD	—	Calculation or prediction
Elimination	CL_int_ (liver)	μl/min/mg protein	*In vitro* experimentation^c^
	CL_int_ (liver)	μl/min/10^6^ cells	*In vitro* experimentation^d^
	*V*_max_, *K*_m_, and fu_inc_ (liver)	pmol/min/mg protein, μM	*In vitro* experimentation^c^
	*V*_max_, *K*_m_, and *f*_u,inc_ (liver)	pmol/min/10^6^ cells, μM	*In vitro* experimentation^d^
	CL_int_ (intestine)	μl/min/mg protein	*In vitro* experimentation^e^
	CL_int_ (intestine)	μl/min/g intestine	*In vitro* experimentation^f^
	*V*_max_, *K*_m_, and *f*_u,inc_ (intestine)	pmol/min/mg protein, μM	*In vitro* experimentation^e^
	*V*_max_, *K*_m_, and *f*_u,inc_ (intestine)	pmol/min/g intestine, μM	*In vitro* experimentation^f^

*In vitro* measurement of

a Ionization equilibrium constant.

b Include Caco-2 cell permeability experiment, MDCK II cell permeability experiment, and PAMPA (parallel artificial membrane permeability assay) experiment.

c Liver microsome or liver S9 fraction (a post mitochondrial supernatant model containing both the microsomal enzymes and cytosolic fractions of the cell).

d Hepatocyte experimentation.

e Intestinal microsome or intestinal S9 fraction.

f Intestinal slice.

The drug plasma concentration–time profile and PK parameters {areas under the concentration–time curve to last time point [AUC_(0–t)_], peak plasma concentration (C_max_), and time to reach C_max_ (T_max_)} after orally administered single-dose of model drugs were predicted. Meanwhile, the PK process of some model drugs administrated with various doses was simulated.

#### 2.2.1 Physiologically Based Pharmacokinetic Model for Phenacetin

The *in vitro* properties of phenacetin collected from various literatures and databases are listed in Supplementary Table S1. The reported *f*_u_ values ranged from 0.145 to 0.5. The metabolism of phenacetin by CYP1A2 is biphasic in microsome experiments (Kahn et al., 1987). Since the hepatic CL_int_ collected from reported microsome experiments varied widely (0.086–100 μl/min/mg protein), we selected the median value of *f*_u_ (0.3225) and hepatic CL_int_ (27 μl/min/10^6^ cells, 20.7–78 μl/min/10^6^ cells) from hepatocyte experiments as input values in our model. The *in vitro* data parameterized in phenacetin PBPK model are tabulated in Table 2. The simulated results of phenacetin after oral administration of 20, 10, and 5 mg/kg in rats were evaluated.

**TABLE 2:** Input parameters in the PBPK models.

Parameter/Compound	Phenacetin	Tolbutamide	Omeprazole	Metoprolol	Chlorzoxazone	Nifedipine	Baicalein
MW (g/mol)	179.2	270.35	345.42	267.4	169.56	346.3	270.24
log*P*_o:w_	1.58	2.34	2.23	2.06	1.6	2.2	1.7
Compound type	Neutral	Monoprotic acid	Ampholyte	Monoprotic base	Monoprotic acid	Monoprotic base	Monoprotic acid
p*K*_a_1	/	5.16	8.8	9.7	8.3	2.82	5.4
p*K*_a_2	/	/	4.2	/	/	/	/
B/P	1	1.33	0.66	1	1.22	0.59	1.27
*f*_u_	0.32	0.048	0.19	0.86	0.27	0.038	0.054
PSA (Å^2^)	38.33	80.65	86.7	50.7	38.3	110	87
HBD	1	2	1	2	1	1	3
CL_int_ (LM) (µl/min/mg protein)	27 (μl/min/10^6^ cells)	4.7	158	32	14.9	139	436
CL_int_ (IM) (µl/min/mg protein)	/	/	/	10.5	/	6.4	298
*V*_max_ (IM) (pmol/min/mg)	0.25	/	780	/	/	/	/
*K*_m_ (IM) (µM)	56.7	/	6.97	/	/	/	/
*f*_u inc_ (IM)	1	/	1	/	/	/	/

“/” means no input value exists.

#### 2.2.2 Physiologically Based Pharmacokinetic Model for Tolbutamide

Tolbutamide is mainly eliminated by CYP2C9 in human liver, and by CYP2C6 and CYP2C11 in rats to produce hydroxyl tolbutamide. *f*_u_ of tolbutamide varied from 0.0201 to 0.268 (listed in Supplementary Table S1), with the calculated median value to be 0.048. The CL_int_ ranged between 2.72 and 8.10 μl/min/mg protein from various literatures and the median of 4.7 μl/min/mg protein was applied in the PBPK model. The same process was performed for other parameters in Table 2. The PK parameters of tolbutamide at 50 mg/kg in rats were predicted.

#### 2.2.3 Physiologically Based Pharmacokinetic Model for Omeprazole

As shown in Supplementary Table S1, various *in vitro* data were collected and the deviation of the reported *in vitro* parameters from different sources was slight. Omeprazole is rapidly absorbed in rats and the elimination is almost entirely through hepatic and intestinal metabolism *via* CYP2C19 (Regårdh et al., 1985; Paul, 1991). The penetration of omeprazole into the red cells is low with the value of B/P at 0.6∼0.8 and the *f*_u_ is about 15% in rat plasma. Similarly, the median values of parameters were calculated for model construction (Table 2). Plasma concentration over time in rats following oral administration of omeprazole at 10, 20, and 40 mg/kg was predicted, respectively.

#### 2.2.4 Physiologically Based Pharmacokinetic Model for Metoprolol

The elimination of metoprolol *in vivo* is mainly through liver CYP2D6 in human, leading to extensive first-pass effect and low bioavailability. In addition to CYP2D6, CYP3A also participates in the metoprolol metabolism in rats. As shown in Supplementary Table S1, *f*_u_ ranged from 0.8 to 0.925, the range of CL_int_ [liver microsomes (LM)] was 17.1–59.9 μl/min/mg protein and the range of CL_int_ [intestine microsomes (IM)] was 7.37–14.7 μl/min/mg protein. The median value of varied parameters was calculated as input parameters in Table 2. The disposition process of metoprolol in rats after oral administration of 2.5, 5, and 20 mg/kg was predicted.

#### 2.2.5 Physiologically Based Pharmacokinetic Model for Chlorzoxazone

Chlorzoxazone is the probe substrate of CYP2E1. As shown in Supplementary Table S1, the value of *f*_u_ ranged from 0.046 to 0.373 and the CL_int_ was from 5.00 to 38.8 μl/min/mg protein. The value of other parameters collected from different sources was relatively consistent. Similarly, the median value was taken as the input value in Table 2. Since a dose of 50 mg/kg was frequently used in the PK studies of chlorzoxazone in rats, the PK parameters of chlorzoxazone following oral administration of 50 mg/kg were predicted.

#### 2.2.6 Physiologically Based Pharmacokinetic Model for Nifedipine

Nifedipine is mainly metabolized by CYP 3A1/2 in human and CYP 3A4/5 in rats. As shown in Supplementary Table S1, the value of *f*_u_ was 0.01–0.08, and the median value was calculated for input. The clearance of nifedipine was found related to concentration (Iwao et al., 2002). The CL_int_ in the liver was 159 μl/min/mg protein when the concentration was 1–5 μM, which was reduced to 119 μl/min/mg protein when the concentration ranged from 5 to 100 μM and eventually dropped to 10 μl/min/mg protein with concentration higher than 100 μM. The value of CL_int_ of small intestinal metabolism was estimated at 6.4 μl/min/mg protein when the concentration was lower than 5 μM and reduced to 2.8 μl/min/mg protein as the concentration was increased to 100 μM. Since the concentration in the liver was unlikely to be higher than 100 μM, the median of hepatic CL_int_ was calculated to be 139 μl/min/mg protein with the ignorance of the lowest value at 10 μl/min/mg protein. The median of CL_int_ of small intestinal metabolism was also calculated for the PBPK model (Table 2). The PK parameters of nifedipine in rats after oral administration of 3, 5, and 6 mg/kg were predicted.

#### 2.2.7 Physiologically Based Pharmacokinetic Model for Baicalein

Baicalein, a bioactive flavonoid presented in the root of *Scutellaria baicalensis*, is isolated from traditional Chinese medicine “Huang Qin.” Baicalein was reported to be subjected to extensive first-pass metabolism due to the conjugated process by UGT in the liver and intestine. Few *in vitro* data were reported (listed in Supplementary Table S1), and the median value was calculated for model construction (shown in Table 2). The PK parameters of baicalein in rats after oral administration of 121 mg/kg were predicted.

### 2.3 Pharmacokinetic Data

PK parameters (AUC_(0–t)_, C_max_, and T_max_) of model drugs in rats orally administrated with a different single-dose of the drugs were assembled from the literature. The AUC_(0–t)_, C_max_, and T_max_ units were unified as μg·h/ml, μg/ml, and h, respectively. The observed drug plasma concentration–time data were extracted from concentration–time course curves using the GetData software (version 2.24, http://getdata-graph-digitizer.com). Multiple records of PK parameters of model drugs were summarized in Supplementary Table S2, showing significant variations on PK parameters, with those of reasonable trend from different doses selected to validate prediction accuracy. For example, it was found that the PK parameters of tolbutamide from the literature varied considerably. Interestingly, the values of C_max_ and AUC_(0–t)_ at high doses were lower than low doses for some records (C_max_: 91.1–151 μg/ml at 20 mg/kg vs. 40.46 μg/ml at 30 mg/kg, AUC_(0–t)_: 761.7217–1,393 μg·h/ml at 20 mg/kg vs. 183.88 μg·h/ml at 20 mg/kg), and T_max_ also varied greatly (0.89–7.1 h). The relatively consistent PK value at a 50 mg/kg dose was finally selected for the tolbutamide PBPK model accuracy evaluation. Meanwhile, it is difficult to detect baicalein’s plasma concentration because of the complicated *in vivo* disposition, poor bioavailability, and extensive metabolism, leading to a considerable variation among the PK parameters. The ones after oral administration of 121 mg/kg were selected due to relatively consistent values (shown in Supplementary Table S2). Moreover, it was found to be partly due to the species, age, and gender differences of the rats and formulations. For example, it was reported that the AUC_(0–t)_ and C_max_ of metoprolol were 5–7 times higher and 2 times higher in DA (Dark Agouti) rats and Sprague–Dawley rats, respectively, than those in Wistar rats after oral administration of 5 mg/kg (Belpaire et al., 1990; Komura and Iwaki, 2005; Wang et al., 2014; Ma et al., 2015; Sun et al., 2017). The AUC_(0–t)_ of baicalein was 11.7 times higher after oral administration of baicalein–nicotinamide nano-cocrystals than coarse powder, 7.1 times higher than that of baicalein nanocrystals, and 1.8 times higher than that of baicalein–nicotinamide cocrystals (Pi et al., 2019). As a result, our PBPK model was constructed using male Sprague–Dawley rats as the model animal with coarse powder form selected.

### 2.4 Sensitivity Analysis

**FIGURE 1:**
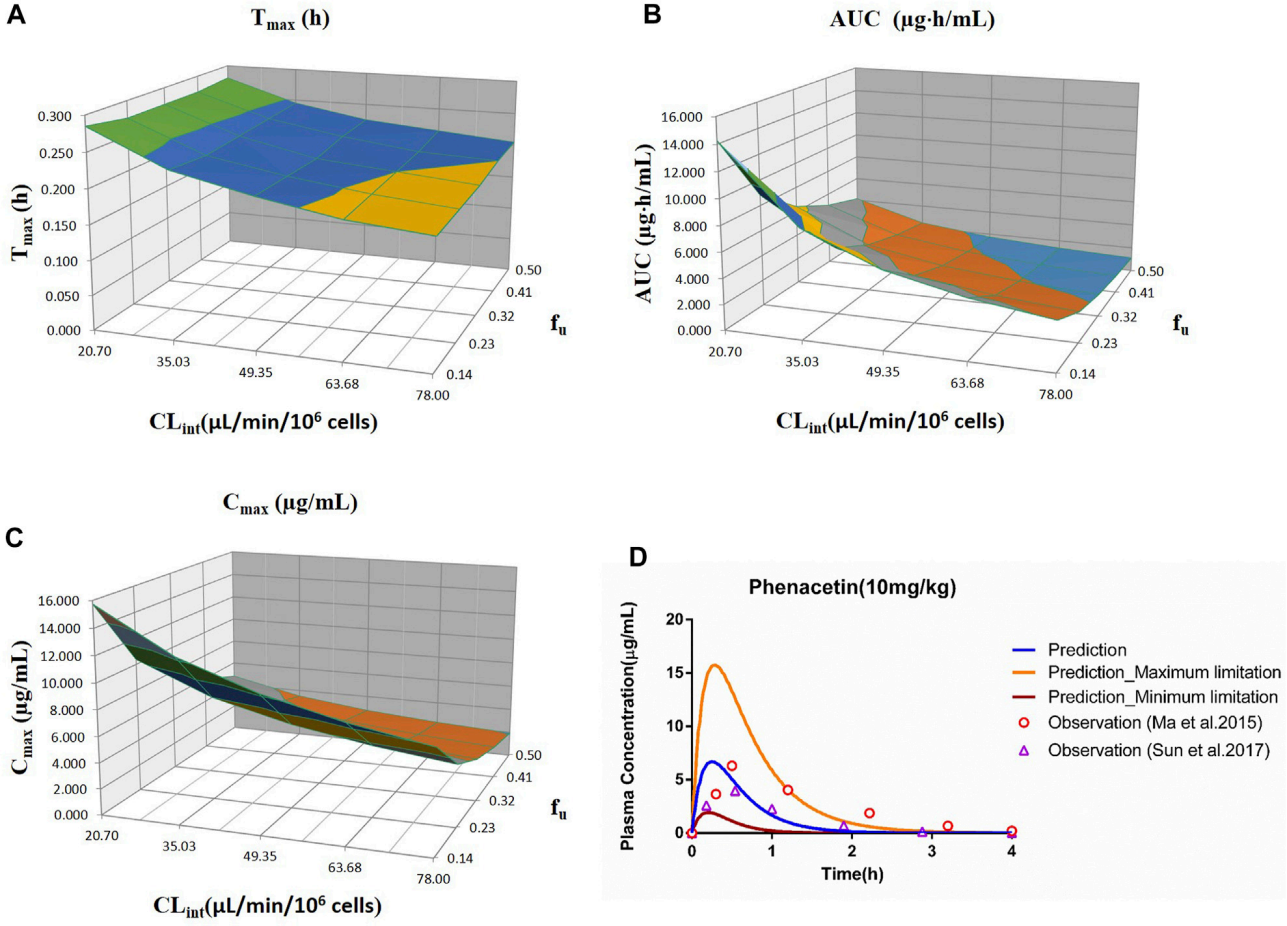
Influence of *f*_u_ and CL_int_ (hepatocytes) on the predicted T_max_ **(A)**, AUC_(0–t)_ **(B)**, C_max_ **(C)**, and plasma concentration–time profile **(D)** of the phenacetin PBPK model. Each color in the 3D plots in **(A–C)** represents a prediction range.

**FIGURE 2:**
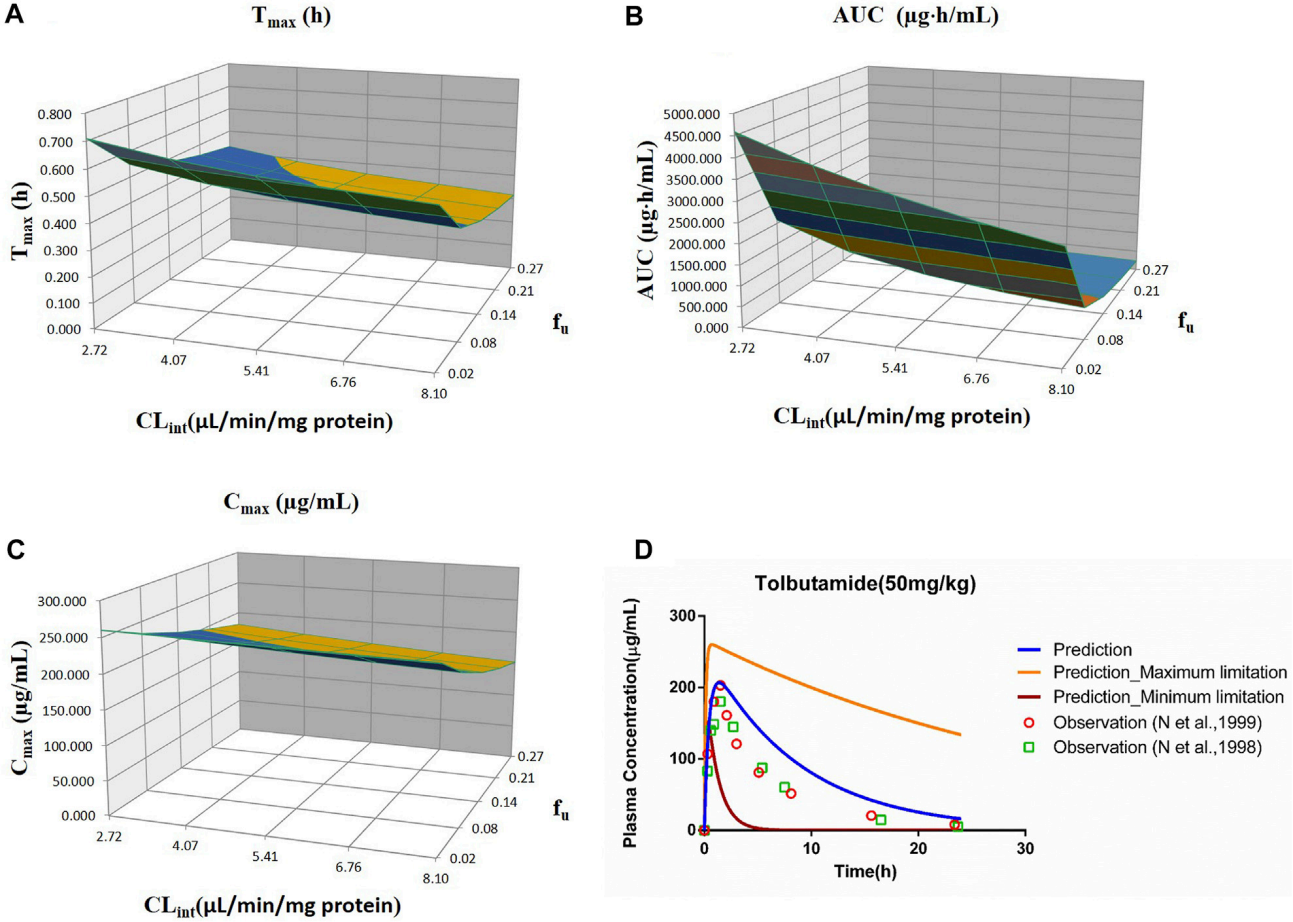
Influence of *f*_u_ and CL_int_ (LM) on the predicted T_max_ **(A)**, AUC_(0–t)_ **(B)**, and C_max_ **(C)** and plasma concentration–time profile **(D)** of tolbutamide. Each color in the 3D plots in **(A–C)** represents a prediction range.

**FIGURE 3:**
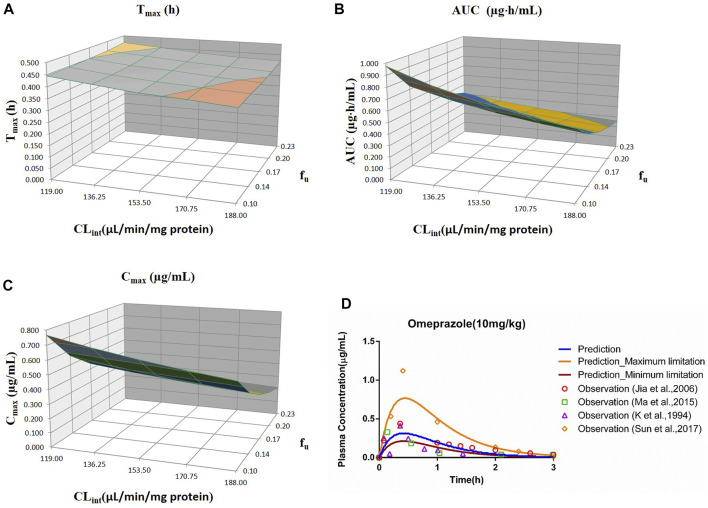
Influence of *f*_u_ and CL_int_ (LM) on the predicted T_max_ **(A)**, AUC_(0–t)_ **(B)**, and C_max_ **(C)** and plasma concentration-time profile **(D)** of the omeprazole PBPK model. Each color in the 3D plots in **(A–C)** represents a prediction range.

**FIGURE 4:**
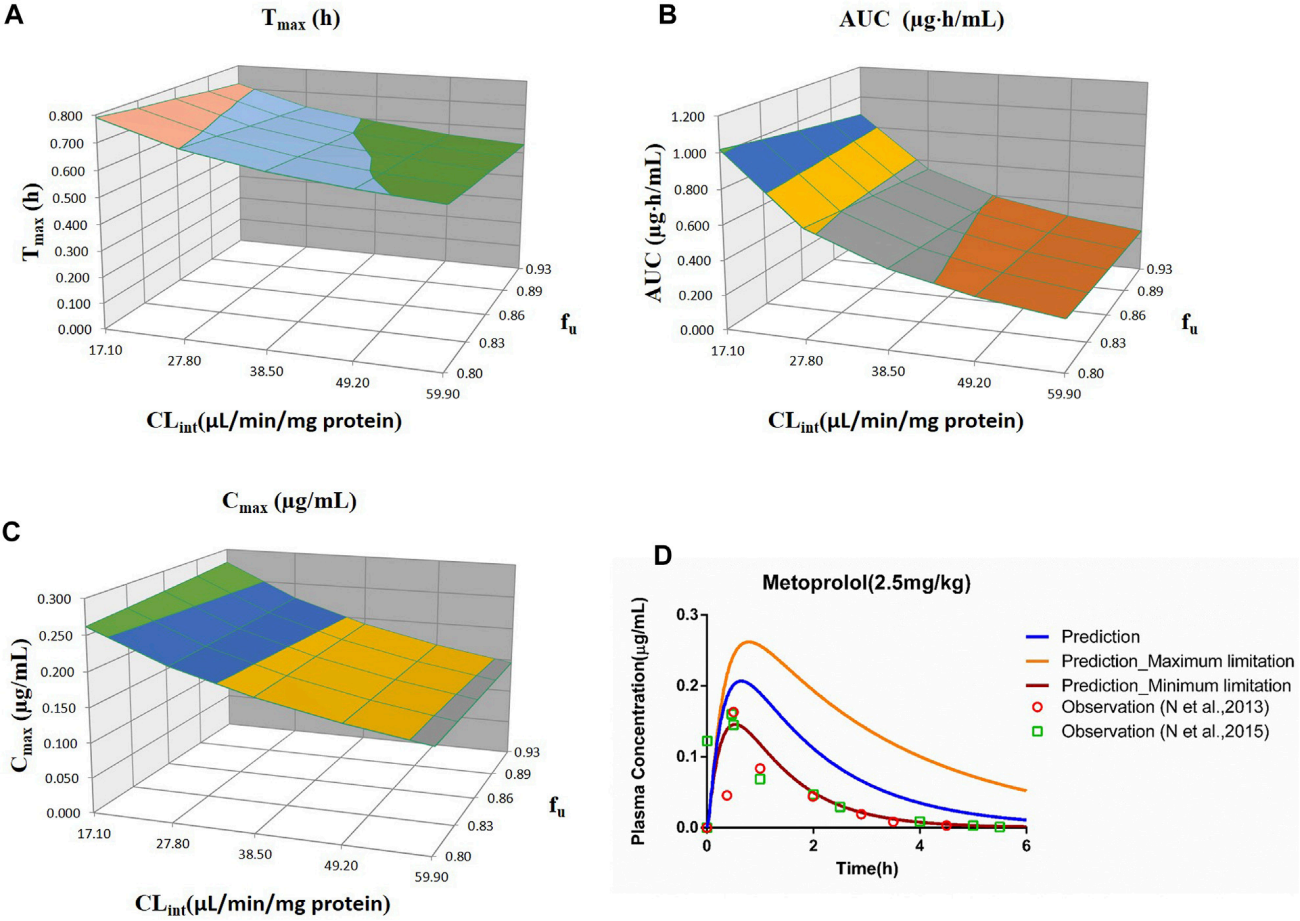
Influence of *f*_u_ and CL_int_ (LM) on the predicted T_max_ **(A)**, AUC_(0–t)_ **(B)**, C_max_ **(C)**, and plasma concentration–time profile **(D)** of the metoprolol PBPK model. Each color in the 3D plots in **(A–C)** represents a prediction range.

**FIGURE 5:**
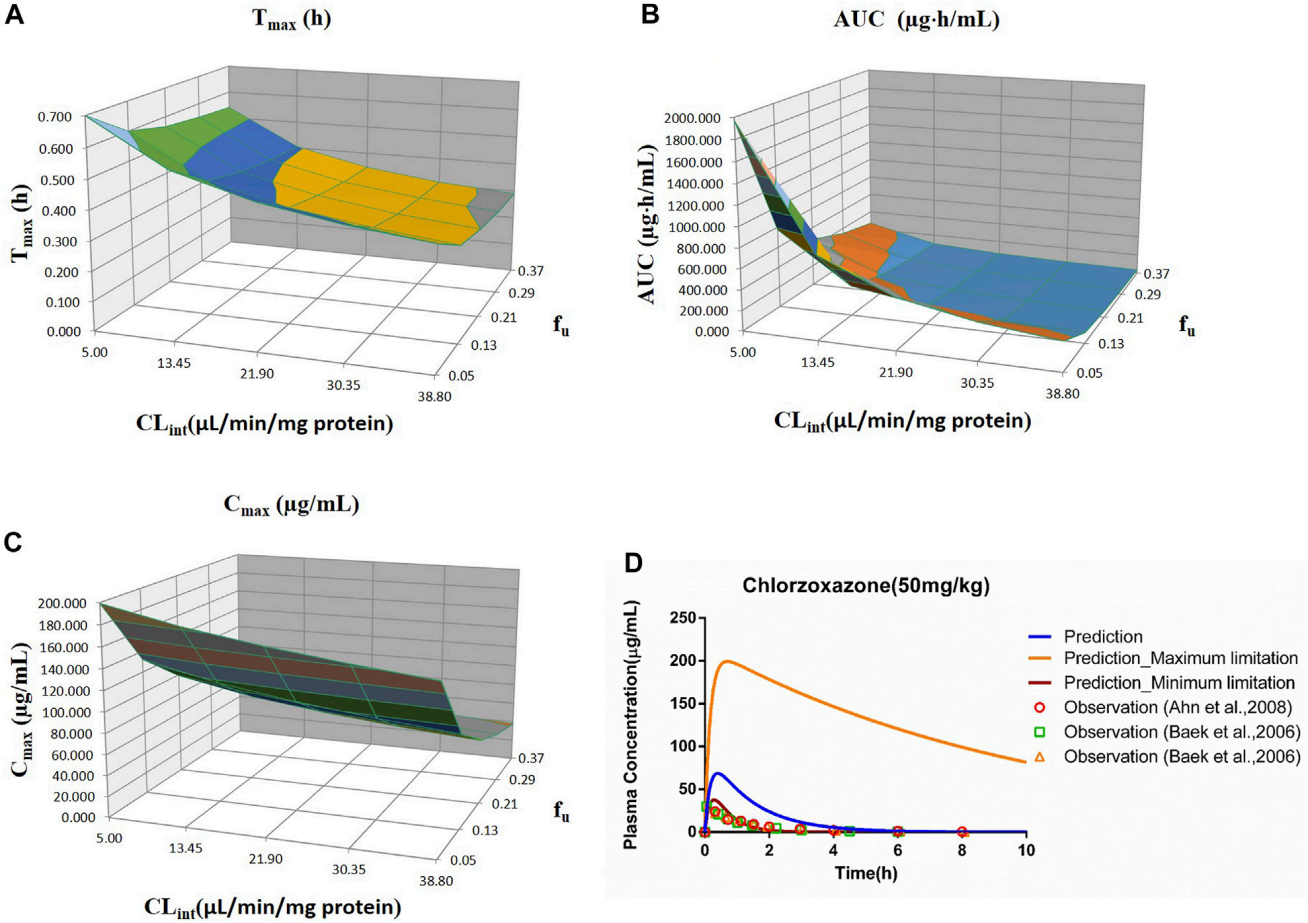
Influence of *f*_u_ and CL_int_ (LM) on the predicted T_max_ **(A)**, AUC_(0–t)_ **(B)**, C_max_ **(C)**, and plasma concentration–time profile **(D)** of the chlorzoxazone PBPK model. Each color in the 3D plots in **(A–C)** represents a prediction range.

**FIGURE 6:**
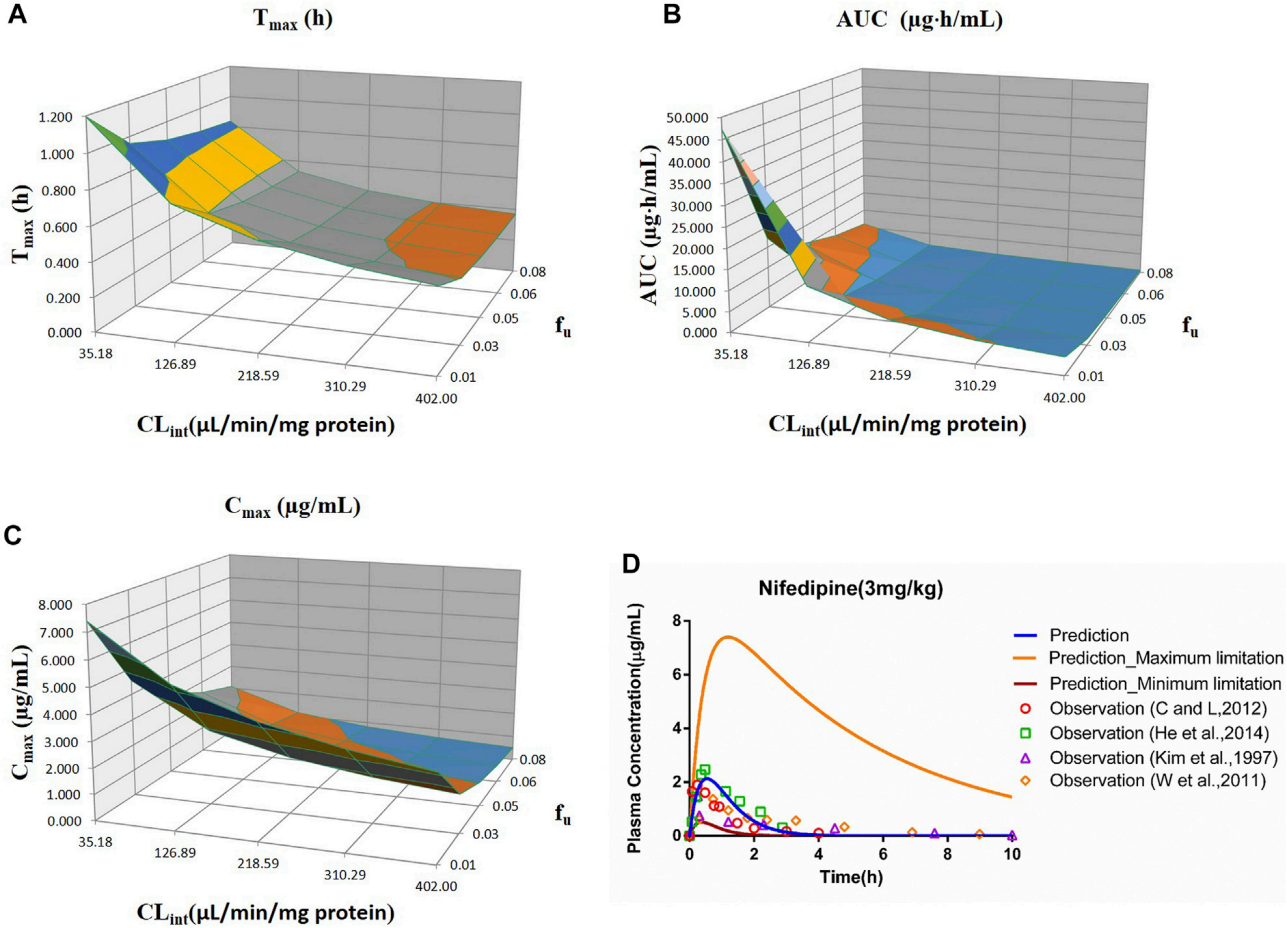
Influence of *f*_u_ and CL_int_ (LM) on the predicted T_max_ **(A)**, AUC_(0–t)_ **(B)**, C_max_ **(C)**, and plasma concentration–time profile **(D)** of the nifedipine PBPK model. Each color in the 3D plots in **(A–C)** represents a prediction range.

**FIGURE 7:**
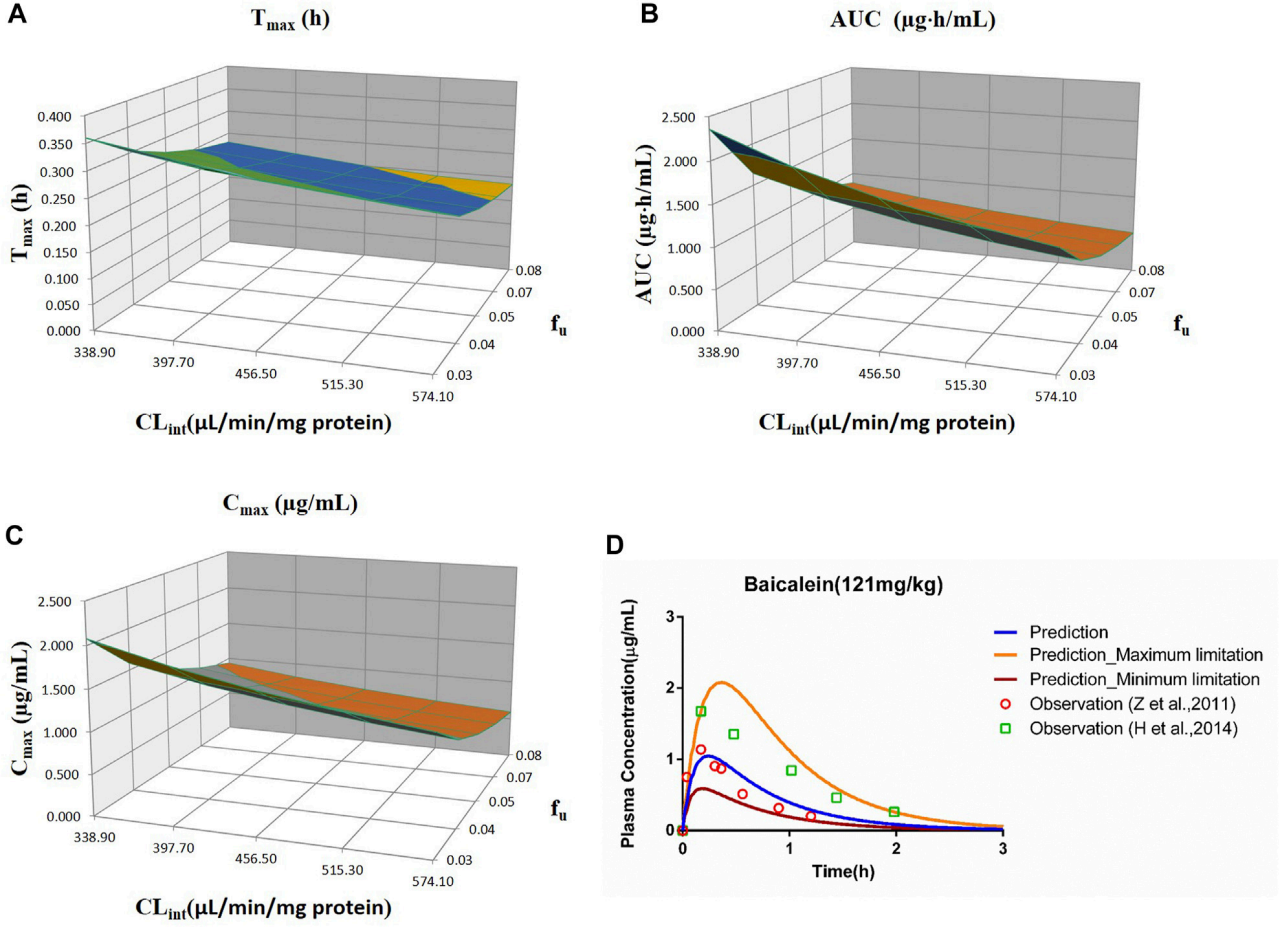
Influence of *f*_u_ and CL_int_ (LM) on the predicted T_max_ **(A)**, AUC_(0–t)_ **(B)**, C_max_ **(C)**, and plasma concentration–time profile **(D)** of the baicalein PBPK model. Each color in the 3D plots in **(A–C)** represents a prediction range.

As shown in Supplementary Table S1, physical chemistry properties such as log*P*_o:w_, compound type, p*K*_a_, and B/P and the predicted or calculated properties (MW, PSA, and HBD) were commonly consistent, while the *f*_u_ and CL_int_ (LM or hepatocytes) values obtained from the literature varied. To further explore the reliability of the PBPK model, the effect of *f*_u_ and CL_int_ (LM or hepatocytes) was investigated. The sensitivity analysis of *f*_u_ and CL_int_ (LM or hepatocytes) was determined *via* comparing the predicted results of PBPK models, which were constructed using the range values of *f*_u_ and CL_int_ (LM or hepatocytes) with all the other factors maintained constant. The uncertainty range of the PBPK model was defined as the predicted range of the PK parameters in the sensitivity analysis. The sensitivity results were plotted *via* Simcyp and showed in Figures 1–7. In order to make the 3D plots in A–C in Figures 1–7 more clearly distinguished, the predicted values were divided into 5–8 range values at the same interval, and each range was displayed as a color. Since the colors are numerous and have no special meaning, they are not specifically listed in the figures.

### 2.5 Model Validation

To verify the prediction accuracy of the PBPK model, we compared the predicted parameters with the experimental data, and fold error was introduced to measure the deviation. The fold error is the ratio between the predicted PK parameters and the corresponding observed values (shown in Eqs. 1, 2). The accuracy of the prediction results increases as the fold error decreases. The simulation is considered acceptable with a fold error of less than 2 (De Buck et al., 2007; Yamazaki et al., 2011).

$$fold\;error=\frac{observed\;parameter}{predicted\;parameter};if\;observed\;value>predicted\;value$$ (1)

$$fold\;error=\frac{predicted\;parameter}{observed\;parameter};if\;predicted\;value>observed\;value.$$ (2)

### 2.6 Model Performance

R^2^, mean absolute error (MAE), and root mean squared error (RMSE) were applied to evaluate the overall performance of the PBPK model. The equations are presented as follows (Eqs. 3–5). The lower the value of MAE and RMSE and the closer of R^2^ to 1, the better the performance of the PBPK model. The performance of the models was plotted using the GraphPad Prism software (version 6).

$$R^{2}=1-\frac{\sum_{i}{\left(x{\;}_{i}-y_{i}\right)}^{2}}{\sum_{i}{\left(x_{i}-\bar{x}\right)}^{2}},$$ (3)

$$MAE=\frac{\sum_{1}^{N}\left|x_{i}-y_{i}\right|}{N},$$ (4)

$$RMSE=\sqrt{\frac{\sum_{1}^{N}{\left(x_{i}-y_{i}\right)}^{2}}{N}}.$$ (5)

x_i_ and y_i_ are the observed and the predicted concentrations, respectively, ‾x is the average of the observed values, and ‾N is the number of data points.

## 3 Results

### 3.1 Physiologically Based Pharmacokinetic Model-Predicted Results

PBPK models for probe drugs (phenacetin, tolbutamide, omeprazole, metoprolol, chlorzoxazone, nifedipine, and baicalein) were constructed using *in vitro* parameters (MW, log*P*_o:w_, compound type, p*K*_a_, B/P, *f*_u_, PSA, HBD, CL_int,_ *V*_max_, *K*_m_, and *f*_u,inc_). The predicted results of each drug were presented later, and the PK curves are displayed in Supplementary Figures S1–S7.

#### 3.1.1 Phenacetin

The predicted results of the phenacetin PBPK model are shown in Table 3. Although the predicted C_max_ was slightly higher than the observed one with the fold error of 1.93 at 5 mg/kg, the fold error values were all within the threshold of 2, indicating good simulation performance. Meanwhile, the predicted data were slightly higher than the observed ones at 5 mg/kg, while other observed points were around the predicted values at 10 and 20 mg/kg (Supplementary Figure S1). It could be concluded that the experimental CL_int_ value used in the model was underestimated at 5 mg/kg.

**TABLE 3:** Comparison between the predicted and the observed PK parameters of the phenacetin PBPK model.

	Dose	T_max_ (h)	C_max_ (μg/ml)	AUC_(0–t)_ (μg•h/ml)	Reference
5 mg/kg	Prediction	0.25	3.34	2.47	—
	Observation	0.33–0.50	1.73	1.52	Zhou et al. (2014)
	Fold error	1.31–2.00	1.93	1.62	—
10 mg/kg	Prediction	0.25	6.69	4.95	—
	Observation 1	0.50	6.28 ± 1.94	9.79 ± 3.58	Ma et al. (2015)
	Fold error	1.98	1.07	1.98	—
	Observation 2	—	3.94 ± 0.81	4.69 ± 0.86	Sun et al. (2017)
	Fold error	—	1.70	1.06	—
20 mg/kg	Prediction	0.25	13.37	9.90	—
	Observation	0.25	9.00	13.12	Welch et al. (1976)
	Fold error	1.01	1.49	1.33	—

#### 3.1.2 Tolbutamide

The prediction results are listed in Table 4. The fold error values were all less than 2 with the observed data around the predicted curve (Supplementary Figure S2), suggesting acceptable prediction accuracy.

**TABLE 4:** Comparison between the predicted PK parameters of the tolbutamide PBPK model and the observed PK parameters at 50 mg/kg.

50 mg/kg p.o.	T_max_ (h)	C_max_ (μg/ml)	AUC_(0–t)_ (μg•h/ml)	Reference
Prediction	1.38	206.74	1965.99	—
Observation 1	0.91 ± 0.37	232.00 ± 35.00	1,309.00 ± 40.00	Nishimura et al. (1999)
Fold error	1.51	1.12	1.50	—
Observation 2	1.42 ± 0.56	176.00 ± 37.20	1,228.00 ± 153.00	Nishimura et al. (1998)
Fold error	1.03	1.17	1.60	—

#### 3.1.3 Omeprazole

The predicted PK parameters of the omeprazole PBPK model were compared with the multiple records of experimental parameters (shown in Table 5). Although a few predicted values were slightly lower than the observed ones, most fold errors fell within 2, indicating good predicted accuracy. Supplementary Figure S3 showed that most of the predicted points were around the observed concentration–time profile.

**TABLE 5:** Comparison between the predicted PK parameters of the omeprazole PBPK model and the observed PK parameters.

	Dose	T_max_ (h)	C_max_ (μg/ml)	AUC_(0–t)_ (μg•h/ml)	Reference
10 mg/kg	Prediction	0.42	0.31	0.38	—
	Observation 1	0.36 ± 0.22	0.44 ± 0.12	0.59 ± 0.14	Jia et al. (2006)
	Fold error	1.16	1.40	1.55	—
	Observation 2	0.10 ± 0.10	0.33 ± 0.01	0.47 ± 0.13	Ma et al. (2015)
	Fold error	4.20	1.07	1.24	—
	Observation 3	0.21 ± 0.02	0.50 ± 0.12	0.29 ± 0.07	Kazuhide et al. (1994)
	Fold error	2.00	1.60	1.32	—
	Observation 4		1.13 ± 0.18	1.13 ± 0.16	Sun et al. (2017)
	Fold error		3.60	2.97	—
20 mg/kg	Prediction	0.40	0.81	0.96	—
	Observation 1	0.25 ± 0.02	1.43 ± 0.38	0.86 ± 0.15	Kazuhide et al. (1994)
	Fold error	1.58	1.77	1.11	—
	Observation 2	0.25 ± 0.00	1.01 ± 0.17	0.73 ± 0.06	Watanabe et al. (2002)
	Fold error	1.58	1.25	1.31	—
	Observation 3	0.50 ± 0.00	2.01 ± 0.14	1.50 ± 0.08	Singh and Asad (2010)
	Fold error	1.26	2.49	1.57	—
40 mg/kg	Prediction	0.37	2.88	3.26	—
	Observation 1	0.29 ± 0.22	2.11 ± 0.99	1.57 ± 0.54	Lee et al. (2009)
	Fold error	1.27	1.36	2.08	—
	Observation 2	0.17 ± 0.09	2.61 ± 0.55	2.10 ± 0.93	Young et al. (2007)
	Fold error	2.23	1.10	1.55	—
	Observation 3	0.68 ± 0.82	2.66 ± 2.00	3.08 ± 1.44	Lee et al. (2007b)
	Fold error	1.82	1.08	1.06	—
	Observation 4	0.35 ± 0.39	3.30 ± 1.65	2.27 ± 1.07	Lee et al. (2006)
	Fold error	1.06	1.15	1.44	—
	Observation 5	0.27 ± 0.02	2.44 ± 0.63	2.26 ± 0.52	Kazuhide et al. (1994)
	Fold error	1.38	1.18	1.44	—
	Observation 6	0.10 ± 0.06	4.89 ± 1.33	1.98 ± 0.60	Lee et al. (2007c)
	Fold error	3.58	1.70	1.64	—
	Observation 7	0.35 ± 0.32	2.43 ± 1.17	1.92 ± 0.88	Lee et al. (2007a)
	Fold error	1.05	1.18	1.70	—

#### 3.1.4 Metoprolol

The fold errors of the predicted parameters of the metoprolol PBPK model are evaluated in Table 6. The values of fold error in the 10 mg/kg group were less than 2, and the trend of the observed data coordinated the predicted profile (Supplementary Figure S4), indicating good simulation performance. Overall, all predicted concentration curves were slightly higher than the observation points, which is contributed by underestimating distribution and elimination.

**TABLE 6:** Comparison between the predicted PK parameters of metoprolol PBPK models and the observed PK parameters at different doses.

	Dose	T_max_ (h)	C_max_ (μg/ml)	AUC_(0–t)_ (μg•h/ml)	Reference
2.5 mg/kg	Prediction	0.65	0.21	0.51	—
	Observation 1	0.50 ± 0.00	0.16 ± 0.00	0.21 ± 0.02	Nandi et al. (2013)
	Fold error	1.30	1.27	2.39	—
	Observation 2	0.47 ± 0.07	0.16 ± 0.01	0.22 ± 0.02	Nandi et al. (2015)
	Fold error	1.37	1.29	2.28	—
5 mg/kg	Prediction	0.65	0.41	1.01	—
	Observation	0.50	0.40	0.45	Komura and Iwaki (2005)
	Fold error	1.30	1.04	2.25	—
10 mg/kg	Prediction	0.65	0.83	2.02	—
	Observation 1	0.60 ± 0.30	0.57 ± 0.25	1.66 ± 0.58	Ma et al. (2015)
	Fold error	1.08	1.45	1.22	—
	Observation 2	0.70 ± 0.50	0.81 ± 0.28	1.35 ± 0.59	Wang et al. (2014)
	Fold error	1.08	1.02	1.50	—
	Observation 3	—	0.53 ± 0.066	1.49 ± 0.39	Sun et al. (2017)
	Fold error	—	1.56	1.35	—

#### 3.1.5 Chlorzoxazone

The predicted results of the chlorzoxazone PBPK model are tabulated in Table 7. Most fold errors of predicted parameters were larger than 2. The predicted curve was higher and deviated from the observed data (Supplementary Figure S5), which could be related to the underestimation of distribution and elimination. The influence of the two characteristics on the PBPK model will be further explored in the sensitivity analysis.

**TABLE 7:** Comparison between the predicted PK parameters of the chlorzoxazone PBPK model and the observed PK parameters at 50 mg/kg.

50 mg/kg p.o.	T_max_ (h)	C_max_ (μg/ml)	AUC_(0–t)_ (μg•h/ml)	Reference
Prediction	0.40	68.66	124.78	—
Observation 1	0.25 (0.25–0.50)	23.10 ± 8.59	41.67 ± 8.48	Ahn et al. (2008)
Fold error	1.58	2.97	2.99	—
Observation 2	0.14 ± 0.08	31.80 ± 13.10	46.83 ± 16.00	Baek et al. (2006)
Fold error	2.85	2.16	2.66	—
Observation 3	0.10 ± 0.06	30.50 ± 8.17	39.83 ± 4.08	Baek et al. (2006)
Fold error	3.81	2.25	3.13	—

#### 3.1.6 Nifedipine

The predicted results of the nifedipine PBPK model are shown in Table 8. The fold error of C_max_ and AUC_(0–t)_ mainly were within 2, indicating acceptable prediction performance. Although the difference between predicted T_max_ and the observed values was more than 2 times (2.16 times), with a significant individual variation between the measured values of T_max_ (0.08–1.5 h), the fold errors of C_max_ and AUC_(0–t)_ were within 2, indicating good prediction accuracy. As shown in Supplementary Figure S6, the predicted concentration curve was slightly shifted due to the difference in T_max_ at 5 mg/kg, while the observed dots were close to the predicted ones at 3 and 6 mg/kg.

**TABLE 8:** Comparison between the predicted PK parameters of nifedipine PBPK models and the observed PK parameters at different doses.

	Dose	T_max_ (h)	C_max_ (μg/ml)	AUC_(0–t)_ (μg•h/ml)	Reference
3 mg/kg	Prediction	0.54	2.13	3.18	—
	Observation 1	0.25 (0.08–0.50)	2.00 ± 0.68	2.23 ± 0.42	Choi and Lee (2012)
	Fold error	2.16	1.07	1.42	—
	Observation 2	0.47 ± 0.03	2.46 ± 0.29	4.30 ± 0.45	He et al. (2014)
	Fold error	1.14	1.15	1.35	—
	Observation 3	0.25	1.48 ± 0.38	2.84 ± 0.19	Kim et al. (1997)
	Fold error	2.16	1.44	1.12	—
	Observation 4	0.58 ± 0.13	1.68 ± 0.58	3.38 ± 0.60	Wang et al. (2011)
	Fold error	1.07	1.27	1.06	—
5 mg/kg	Prediction	0.54	3.56	5.30	—
	Observation 1	0.38 ± 0.06	1.76 ± 0.20	2.72 ± 0.34	Mutsunobu et al. (2004)
	Fold error	1.42	2.02	1.95	—
	Observation 2	0.25 (0.12–0.50)	1.95 ± 0.26	2.73 ± 0.40	Ikehata et al. (2008)
	Fold error	2.16	1.82	1.94	—
	Observation 3	0.25 (0.12–1.50)	1.96 ± 0.23	3.75 ± 0.63	Ikehata et al. (2008)
	Fold error	2.16	1.81	1.41	—
	Observation 4	0.25 (0.12–1.00)	2.56 ± 0.23	4.38 ± 0.29	Ikehata et al. (2008)
	Fold error	2.16	1.39	1.21	—
6 mg/kg	Prediction	0.54	4.27	6.36	—
	Observation 1	0.38 (0.20–0.57)	5.23 (4.55–6.01)	5.75 (4.72–6.98)	Grundy et al. (1998)
	Fold error	1.41	1.23	1.11	—
	Observation 2	0.28 (0.16–0.40)	5.88 (3.33 ± 10.40)	5.90 (4.73–7.35)	Grundy et al. (1997)
	Fold error	1.94	1.38	1.08	—

#### 3.1.7 Baicalein

The simulation results are shown in Table 9. The predicted curve is shown in Supplementary Figure S7. Although the predicted C_max_ was slightly lower than the observed one, the fold errors were within 2. The observed PK data spread around the predicted profile, suggesting good prediction performance.

**TABLE 9:** Comparison between the predicted PK parameters of the baicalein PBPK model and the observed PK parameters at 121 mg/kg.

121 mg/kg p.o.	T_max_ (h)	C_max_ (μg/ml)	AUC_(0–t)_ (μg•h/ml)	Reference
Prediction	0.24	1.05	0.98	—
Observation 1	0.17 ± 0.00	1.24 ± 0.78	0.79 ± 0.08	Zhang et al. (2011)
Fold error	1.44	1.19	1.25	—
Observation 2	0.17 ± 0.00	1.67 ± 0.85	0.79 ± 0.08	Huang et al. (2014)
Fold error	1.44	1.60	1.25	—

### 3.2 Sensitivity Analysis

The sensitivity analysis of *f*_u_ and CL_int_ (LM or hepatocytes) was evaluated with other factors maintained constant. The variation spans of *f*_u_ and CL_int_ (LM or hepatocytes) from various literatures are listed in Table 10. The uncertainty of the PBPK model-predicted results was estimated based on the range of *f*_u_ and CL_int_ (LM or hepatocytes) obtained from various literatures.

**TABLE 10:** Variation span of the experimental *in vitro* parameters and prediction PK parameters in the sensitivity analysis.

	*f*_u_	CL_int_ (μl/min/mg)	T_max_(h)	C_max_ (μg/ml)	AUC_(0–t)_ (μg•h/ml)
Phenacetin	0.14–0.50	20.70–78.00	0.18–0.28	1.93–15.74	1.10–14.25
Fold range	3.45	3.77	1.56	8.16	12.95
Omeprazole	0.10–0.23	119.00–188.00	0.38–0.46	0.21–0.77	0.26–0.98
Fold range	2.21	1.58	1.21	3.67	3.77
Nifedipine	0.01–0.08	35.18–402	0.36–1.20	0.51–7.40	0.52–47.27
Fold range	8.00	11.43	3.33	14.51	90.90
Chlorzoxazone	0.05–0.373	5.00–38.80	0.29–0.70	37.69–199.27	34.69–1970.18
Fold range	8.11	7.76	2.41	5.29	56.79
Metoprolol	0.80–0.92	17.10–59.90	0.54–0.79	0.15–0.28	0.25–1.02
Fold range	1.16	3.50	1.46	1.87	4.08
Baicalein	0.03–0.08	338.90–574.11	0.19–0.36	0.59–2.08	0.51–2.36
Fold range	2.73	1.69	1.89	3.53	4.63
Tolbutamide	0.02–0.27	2.72–8.10	0.32–0.71	152.72–260.15	231.67–4587.60
Fold range	13.33	2.98	2.22	1.70	19.80

#### 3.2.1 Phenacetin

The sensitivity analysis of *f*_u_ (0.145–0.5) and CL_int_ (hepatocytes: 20.7–78 μl/min/10^6^ cells) for the phenacetin PBPK model was performed at 10 mg/kg (shown in Figure 1). The predicted value of T_max_, C_max_, and AUC_(0–t)_ ranged from 0.18 to 0.28 h, 1.93 to 15.74 μg/ml, and 1.10 to 14.25 μg·h/ml, respectively. Most concentration points are within the uncertainty range.

#### 3.2.2 Tolbutamide

The sensitivity analysis of *f*_u_ (0.0201–0.268) and CL_int_ (LM: 2.72–8.1 μl/min/mg protein) was investigated for the tolbutamide PBPK model at 50 mg/ml (shown in Figure 2). As a result, the predicted T_max_, C_max_, and AUC_(0–t)_ ranged from 0.32 to 0.71 h, 152.72 to 260.15 μg/ml, and 231.67 to 4,587.60 μg·h/ml, respectively. The observed points were in the area between the median prediction curve and the lowest prediction curve (CL_int_ = 8.1 μl/min/mg protein, *f*_u_ = 0.27).

#### 3.2.3 Omeprazole

The range of *f*_u_ (0.105–0.232) and CL_int_ (LM: 119–188 μl/min/mg protein) was applied for the sensitivity analysis of the omeprazole PBPK model at 10 mg/kg (shown in Figure 3). The results showed that the predicted T_max_, C_max_, and AUC_(0–t)_ ranged from 0.38 to 0.46 h, 0.21 to 0.77 μg/ml, and 0.26 to 0.98 μg·h/ml, respectively. As shown in Figure 3D, most observation dots were in the sensitivity prediction range.

#### 3.2.4 Metoprolol

The sensitivity analysis of *f*_u_ (0.80–0.925) and CL_int_ (LM: 17.1–59.9 μl/min/mg protein) was determined for the metoprolol PBPK model at 2.5 mg/kg in Figure 4. The predicted range of T_max_, C_max_, and AUC_(0–t)_ were 0.54–0.79 h, 0.15–0.28 μg/ml, and 0.25–1.02 μg·h/ml, respectively. It is illustrated in Figure 4D that the observation data were close to the lowest predicted curve.

#### 3.2.5 Chlorzoxazone

Sensitivity analysis of the chlorzoxazone PBPK model was performed at 50 mg/kg with the *f*_u_ ranging from 0.046 to 0.373 and CL_int_ (LM) from 5 to 38.8 μl/min/mg protein (shown in Figure 5). The results showed the predicted T_max_, C_max_, and AUC_(0–t)_ ranged from 0.29 to 0.70 h, 37.69 to 199.27 μg/ml, and 34.69 to 1970.18 μg·h/ml, respectively. The observation dots were around the lowest prediction curve (Prediction_Minimum limitation curve), as shown in Figure 5D.

#### 3.2.6 Nifedipine

The sensitivity analysis of *f*_u_ (0.01–0.08) and CL_int_ (LM: 35.18–402 μl/min/mg protein) was investigated for the nifedipine PBPK model at 3 mg/kg (shown in Figure 6). The predicted T_max_, C_max_, and AUC_(0–t)_ ranged from 0.36 to 1.20 h, 0.51 to 7.40 μg/ml, and 0.52 to 47.27 μg·h/ml, respectively. The observation points were centered on the median prediction curve, as shown in Figure 6D.

#### 3.2.7 Baicalein

The sensitivity analysis of *f*_u_ and CL_int_ (LM) was performed with the range from 0.029 to 0.0791 and 338.9 to 574.11 μl/min/mg protein for the baicalein PBPK model at 121 mg/kg (shown in Figure 7 **)**. The results showed the predicted T_max_, C_max_, and AUC_(0–t)_ ranged from 0.19 to 0.36 h, 0.59 to 2.08 μg/ml, and 0.51 to 2.36 μg·h/ml, respectively. As illustrated in Figure 7D, observation dots were within the sensitivity prediction range.

### 3.3 Physiologically Based Pharmacokinetic Model Performance

**FIGURE 8:**
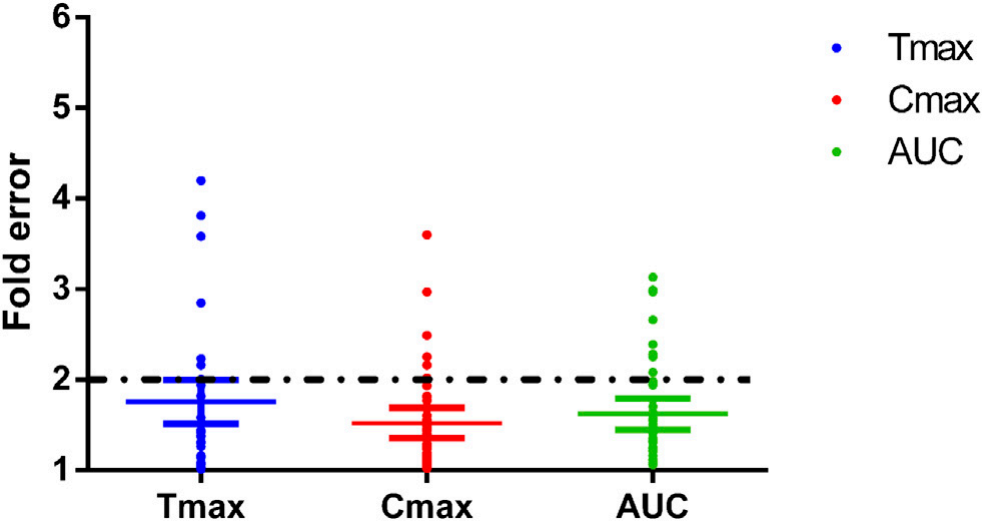
Fold errors of the PK parameters from the PBPK models of phenacetin, tolbutamide, omeprazole, metoprolol, chlorzoxazone, nifedipine, and baicalein. The three lines in each parameter represent mean with 95% confidence interval and the black dotted line across the fold error = 2 represent evaluation criteria.

**FIGURE 9:**
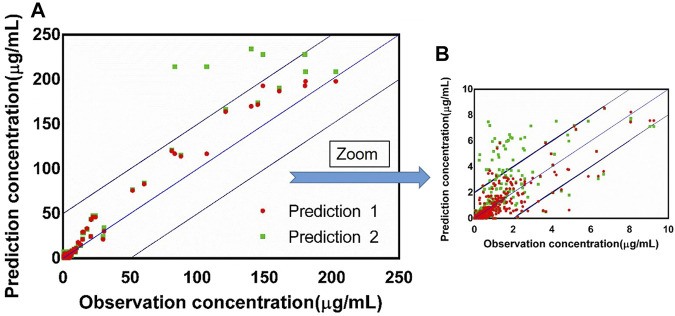
**(A)** Observed concentration vs. predicted concentration (*n* = 390) based on the PBPK model with the absorption module using the PSA and HBD models (prediction 1) and the PBPK model using *P*_app_ from Caco-2 experimentation (prediction 2), blue box: ±20% of the observation concentration; **(B)** graph zoomed at 0–10 μg/ml.

The fold errors are summarized in Figure 8. Furthermore, to evaluate the overall performance of the PBPK model, we compared the prediction concentration data with the observed ones (*n* = 390, shown in Figure 9). Moreover, the prediction performance of the PBPK model with absorption module using PSA and HBD was compared with that using *P*_app_ from *in vitro* experiments (Caco-2 experimentation). The input of *P*_app_ was also applied to the median data of the various values. As shown in Table 11, the model performance using PSA and HBD, with a lower value of MAE and RMSE and higher value of R^2^, was better than using *P*_app_ from Caco-2 experimentation. As displayed in Figure 9B, prediction 1 was closer to the correlation line with most dots within the ±20% of the observed concentration, indicating the overall good performance of PBPK model construction using the PSA and HBD methods.

**TABLE 11:** Performance of the PBPK model with absorption module using PSA and HBD (prediction 1) and PBPK model using *P*_app_ from Caco-2 experimentation (prediction 2).

Performance	R^2^	MAE	RMSE
Prediction 1	0.82	3.30	10.64
Prediction 2	0.58	4.99	16.01

## 4 Discussion

While collecting *in vitro* parameters, we noticed the *f*_u_ and CL_int_ from different sources varied; therefore, the median values were applied to construct the model. A sensitivity analysis was performed to further explore the impact of these two parameters on the model prediction accuracy. The overall predicted accuracy of PBPK models using the median value was good with most fold errors within 2, and the R^2^ between the predicted concentration data and the observed data was more than 0.8 (Figures 8, 9). However, there was an exception that the chlorzoxazone PBPK model using median data was not satisfying with the prediction results, which is 2 times higher than the observed ones. It could be related to the underestimated distribution and clearance. In terms of the sensitivity analysis, the observed points were closest to the lowest prediction curve with the highest CL_int_ (38.8 μl/min/mg protein) and *f*_u_ 0.373 (Moon et al., 2003; Baek et al., 2006). The lowest prediction parameters of T_max_, C_max_, and AUC_(0–t)_ were 0.29 h, 37.69 μg/ml, and 34.69 μg·h/ml, respectively. Moreover, most of the fold errors were within 2 (observation 1–3), indicating good prediction accuracy. Similarly, the prediction result of metoprolol using the median PBPK model was also slightly higher at 2.5 mg/kg, and the observed data was close to the lowest predicted curve. The prediction T_max_, C_max_, and AUC_(0–t)_ of the metoprolol PBPK model using highest experimental CL_int_ (59.9 μl/min/mg) and *f*_u_ (0.93) at 2.5 mg/kg were 0.53 h, 0.15 μg/ml, and 0.25 μg·h/ml, respectively and the fold errors were within 2 (Belpaire et al., 1989; Yoon et al., 2011). These results suggest that applying the PBPK model using *in vitro* parameters in the early screening of candidate compounds is feasible and helpful, and the prediction accuracy of the model is related to the *in vitro* parameters, especially *f*_u_ and CL_int_. It is worth noting that there is a potential correlation between *f*_u_ and CL_int_ under physiological conditions, which likewise may unnaturally increase the variability in predicted values. Furthermore, since the effect of *f*_u_ on CL_int_ might be poorly investigated and difficult to obtain from previous studies, the impact of such correlation on the variability of predicted values remains to be further explored.

In the sensitivity analysis, we summarized the variation span of the experimental *in vitro* parameters and prediction PK parameters in Table 10. Except for nifedipine (fold error of the observed T_max_ was 3.3), the variation of prediction T_max_ in the sensitivity analysis of other drugs was slight, meaning that the experimental difference of *f*_u_ and CL_int_ had little impact on T_max_ compared with C_max_ and AUC_(0–t)_. Compared with phenacetin, omeprazole, metoprolol, and baicalein, the experimental variation of *f*_u_ or CL_int_ of nifedipine, chlorzoxazone, and tolbutamide is relatively large (fold errors of *f*_u_ or CL_int_ > 8), which led to a large variation span in the sensitivity analysis with the fold errors of C_max_ or AUC_(0–t)_ more than 10. Meanwhile, the variation of both *f*_u_ and CL_int_ was higher than 3, resulting in significant fold errors of C_max_ and AUC_(0–t)_ (>8.0). Moreover, inflection points were noted in the change trend graph of *f*_u_ and CL_int_ of phenacetin, nifedipine, chlorzoxazone, and tolbutamide shown in the figure of sensitivity analysis, with the prediction value significantly fluctuating around. Therefore, sensitivity analysis is necessary for model construction. That is to say, when the *in vitro* parameters are close to the inflection point, extra attention should be paid to the PBPK model construction.

In addition to using the PSA and HBD methods to predict the absorption of model drugs in the PBPK model, we also explored the prediction accuracy of the PBPK model using *P*_app_ from *in vitro* experiments such as Caco-2 experimentation. Interestingly, the results showed that the overall prediction performance of PBPK models using the PSA and HBD methods was better. It could be related to defects in the *in vitro* absorption experiments, such as the inability to simulate the dynamic flow of fluids in the body, the lack of mucus layer, the more minor tight junctions, and the thicker unstirred water layer. These may cause the *P*_app_ to not to truly simulate the diffusion of drugs *in vivo* and thus cannot truly predict the drug absorption degree in the body.

It is worth mentioning that the PBPK model using *in vitro* parameters is beneficial to drug development, and the more accurate the value of *in vitro* parameters, the better fit the model. However, the model compounds we applied were BCS (biopharmaceutics classification system) class II drugs, exhibited linear absorption and primarily cleared *via* metabolism. Therefore, our models were appropriate to the PK prediction of chemicals not affected by transporters such as P-gp (p-glycoprotein) efflux transporter. For other compounds with complex drug metabolism characteristics, using *in vitro* parameters alone to construct a PBPK model and the prediction accuracy still require further research and validation. Moreover, in recent years, with the development of computer technology and *in vitro* experiments, the contribution of PBPK models to advancing 3Rs in new drug development is undoubtedly a hot topic of discussion. Since rodents were widely used in preclinical pharmacological experiments, this study used rats as the model animal when constructing PBPK models based on *in vitro* parameters. It is expected that animal experiments can be reduced in the early stage of drug development with the validation of the feasibility and reliability of the models. However, due to the differences in physiological parameters of other species, the construction of their PBPK models needs further exploration and optimization, and due to the complexity of the human body, whether this method can be directly applied to predict the pharmacokinetic characteristics of candidate compounds in the humans and the reliability of the predicted results still need further exploration.

## 5 Conclusion

In our study, bottom–up PBPK models constructed with *in vitro* data were developed, and popular probe substrates were used as model drugs. Most of the fold errors between the observed PK data with the predicted ones were within the threshold of 2, indicating good prediction accuracy. The influence of *in vitro* data was comprehensively analyzed, and results supported that the model accuracy is related to the precision of the *in vitro* data. Moreover, most of the observed PK data is within the uncertainty range, and R^2^ is more than 0.8, indicating the applicability of the PBPK model in the absence of *in vivo* data in the early drug development. In conclusion, a strategy of the bottom–up PBPK model with the quantity of an uncertainty range is constructed, which helps reduce animal experiments and is a good practice of 3Rs in the early screening of candidate compounds.

## Data Availability

The original contributions presented in the study are included in the article/Supplementary Material, further inquiries can be directed to the corresponding authors.
