# Supplementary material for: Application of Physiologically Based Pharmacokinetic Modeling in Preclinical Studies: A Feasible Strategy to Practice the Principles of 3Rs
Source: Front Pharmacol. 2022 May 12;13:895556. doi: 10.3389/fphar.2022.895556 (PMC9133488; doi:10.3389/fphar.2022.895556)
Supplement: Supplementary file 1 [file DataSheet1.DOCX]

Supplementary Material

## Supplementary Figures


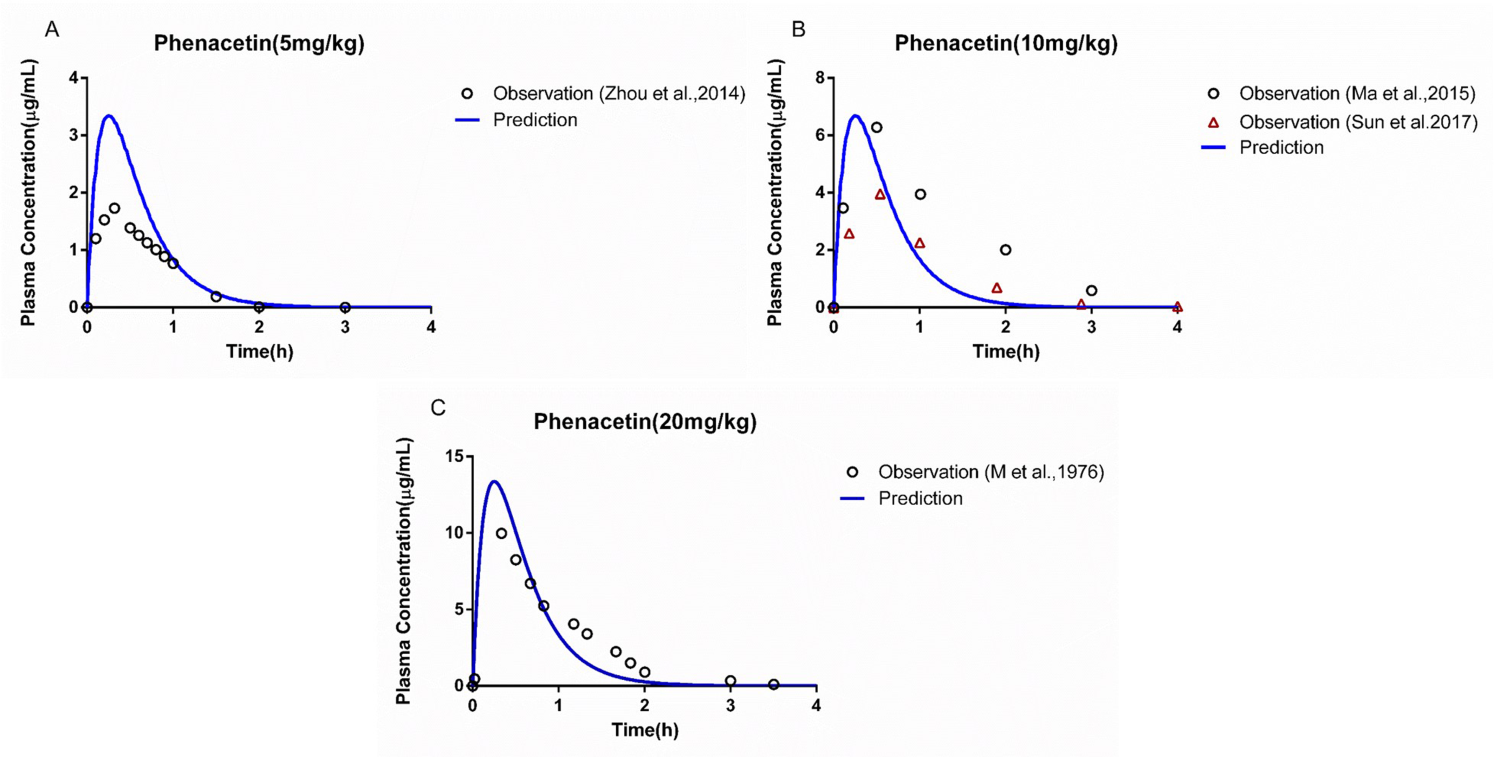


**Supplementary Figure 1.** Comparison of the observed plasma concentration-time data with the simulated plasma concentration-time profile of phenacetin PBPK models dosing at 5 mg/kg (A), 10 mg/kg (B) and 20 mg/kg (C).





**Supplementary Figure 2.** Comparison of the predicted PK curve from tolbutamide PBPK model with the observed plasma concentration-time data at the dose of 50 mg/kg.


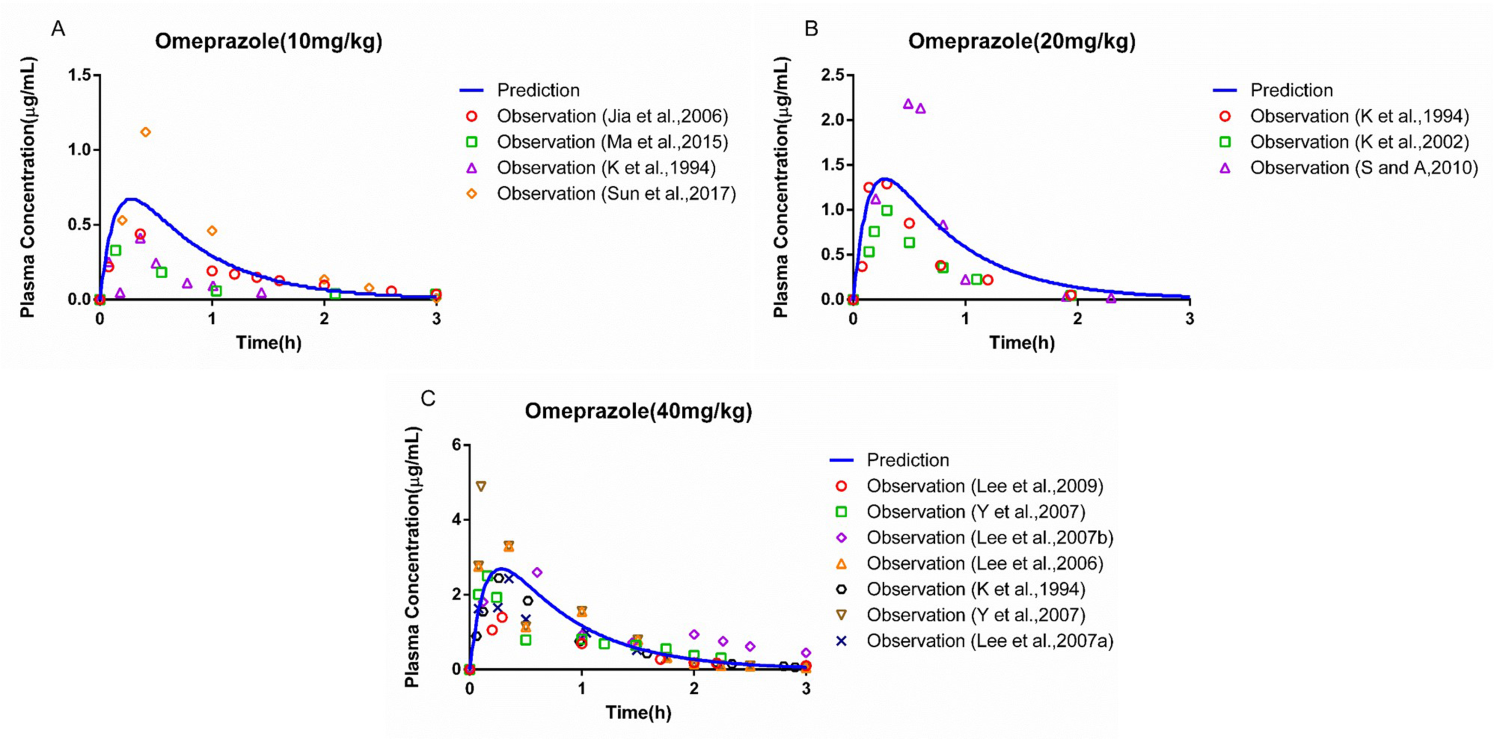


**Supplementary Figure 3.** Comparison of the predicted PK curve from omeprazole PBPK model with observed plasma concentration-time data at a dose of 10 mg/kg (A), 20mg/kg (B) and 40 mg/kg (C ).


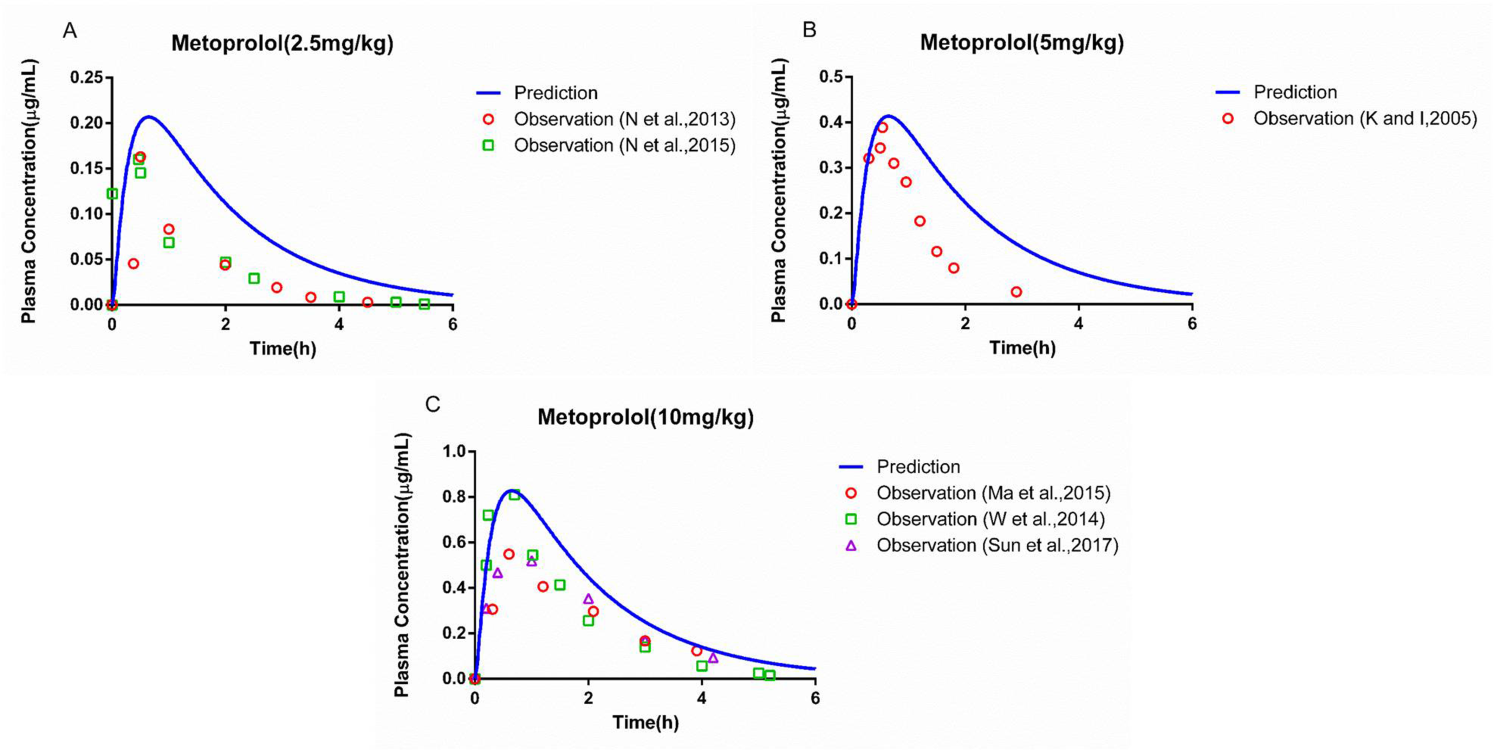


**Supplementary Figure 4.** Comparison of the predicted PK curve from metoprolol PBPK model with the observed plasma concentration-time data at the dose of 2.5 mg/kg (A), 5mg/kg (B) and 10 mg/kg (C).





**Supplementary Figure 5.** Comparison of the predicted PK curve from chlorzoxazone PBPK model with the observed plasma concentration-time data at the dose of 50 mg/kg.


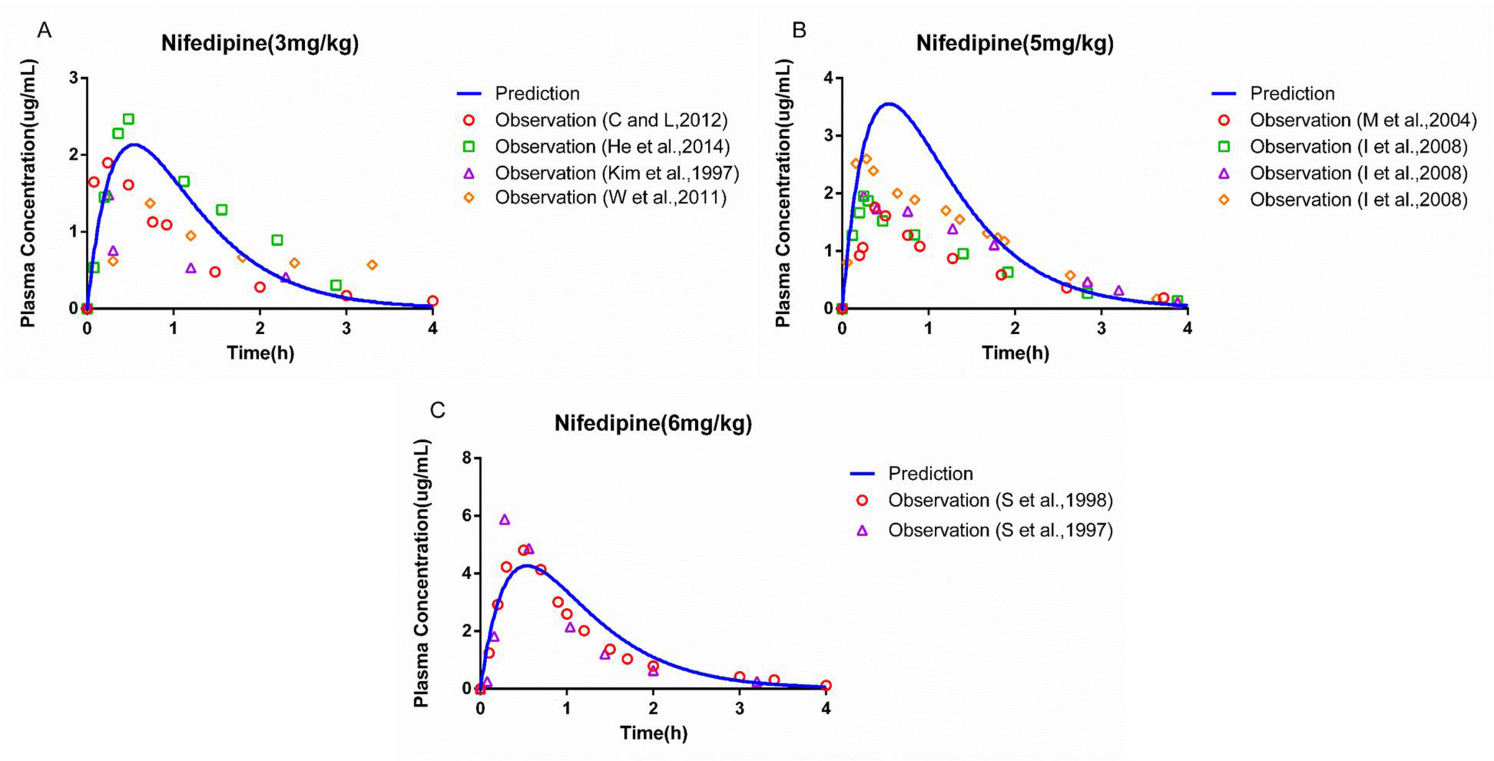


**Supplementary Figure 6.** Comparison of the predicted PK curve from nifedipine PBPK model with observed plasma concentration-time data at the dose of 3 mg/kg (A), 5mg/kg (B) and 6 mg/kg (C).





**Supplementary Figure 7.** Comparison of the predicted PK curve from baicalein PBPK model with the observed plasma concentration-time data at the dose of 121 mg/kg.
